# Association between physical activity, grip strength and sedentary behaviour with incidence of malignant melanoma: results from the UK Biobank

**DOI:** 10.1038/s41416-021-01443-5

**Published:** 2021-05-31

**Authors:** Andrea Weber, Michael F. Leitzmann, Anja M. Sedlmeier, Hansjörg Baurecht, Carmen Jochem, Sebastian Haferkamp, Sebastian E. Baumeister

**Affiliations:** 1 Department of Epidemiology and Preventive Medicine, University of Regensburg, Regensburg, Germany; 2 Department of Dermatology, University Hospital Regensburg, Regensburg, Germany; 3 Institute of Health Services Research in Dentistry, University of Münster, Münster, Germany

**Keywords:** Melanoma, Cancer epidemiology, Risk factors

## Abstract

**Background:**

Physical activity has been positively related to malignant melanoma. However, that association may be confounded by ultraviolet radiation (UV), a variable closely related to both outdoor physical activity and malignant melanoma. We examined physical activity, grip strength and sedentary behaviour in relation to risk of malignant melanoma, accounting for relevant confounders using data from a prospective cohort study.

**Methods:**

In 350,512 UK Biobank participants aged 38–73 years at baseline, physical activity was assessed with a modified version of the International Physical Activity Questionnaire Short Form, grip strength was measured with a hand dynamometer, and sedentary behaviour was recorded with three specific questions. Multivariable hazard ratios (HR) and corresponding 95% confidence intervals (CI) were estimated using Cox proportional hazards regression.

**Results:**

During 7 years of follow-up, 1239 incident malignant melanoma diagnoses were recorded. Physical activity and sedentary behaviour were unrelated to malignant melanoma (HRs 1.01 (95% CI 0.95–1.07) and 1.04 (95% CI 0.97–1.12), respectively), and the initially positive association with grip strength in the basic model (HR 1.23, 95% CI 1.08–1.40) was attenuated after full adjustment (HR 1.10, 95% CI 0.96–1.26).

**Conclusion:**

Physical activity, grip strength and sedentary behaviour are not associated with malignant melanoma risk.

## Background

Physical activity is an established protective factor against risk for various cancers, with the strongest evidence available for colon and breast cancers.^1,2^ Grip strength is a surrogate measure of muscular strength,^3^ a component of physical fitness,^4^ and has also been shown to be inversely associated with colorectal, breast and lung cancers.^5^ Conversely, there is accruing evidence for a positive association between sedentary behaviour and cancers of the colon, breast and endometrium.^6^ Despite biological plausibility that regular exercise protects against malignant melanoma (e.g. through enhanced immune function, decreased chronic inflammation and increased DNA repair^7–9^), prospective cohort studies have consistently shown a positive relationship between physical activity and risk of malignant melanoma.^7^ However, that association could be due to a distorting link between outdoor physical activity and sun exposure.^7,10^ Intermittent exposure to UV radiation (solar and artificial) as well as sunburns are strong risk factors for malignant melanoma but skin phototype also plays an important modifying role and must be taken into account.^11–13^ To the best of our knowledge, the current study is the first to present associations between grip strength, sedentary behaviour and risk of malignant melanoma. Grip strength is determined by environmental and lifestyle factors^14^ as well as genetics (56% heritability for isometric grip strength^15^).

As the incidence of malignant melanoma is increasing globally,^16,17^ possibly due to an increase in long-distance travel and usage of tanning devices,^13^ it is of great importance to identify modifiable risk factors and risk markers, and to investigate whether physical activity, muscular fitness or sedentary behaviour have the potential to influence or predict the development of malignant melanoma. We used prospective data from the UK Biobank with detailed individual-level information on UV exposure and UV sensitivity as well as other relevant confounders to assess the relations of physical activity, grip strength and sedentary behaviour to subsequent risk of malignant melanoma.

## Methods

### Study population and data collection

**Fig. 1:**
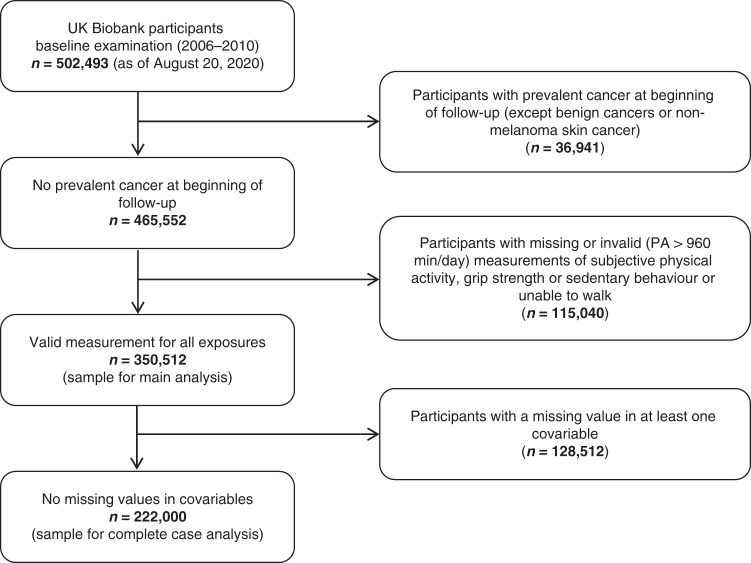
Flow chart of UK Biobank participants. This figure represents the sample selection for the analysis of physical activity (PA), grip strength, sedentary behaviour and malignant melanoma associations.

Between 2006 and 2010, the population-based cohort study UK Biobank examined over 500,000 men and women in 22 study centers in England, Wales and Scotland. Participants were aged 38–73 years and were recruited from different socioeconomic backgrounds.^18^ At baseline and during follow-up visits, various examinations and interviews were conducted and biologic samples were obtained by trained personnel.^18^ The UK Biobank was approved by the North West Multi-centre Research Ethics Committee and written confirmed consent was provided by all participants.^18^ Of the 502,493 available UK Biobank participants (as of August 20, 2020), 36,941 participants with prevalent cancer other than non-melanoma skin cancer at baseline were excluded. After further excluding participants who reported they were unable to walk, those with a missing value for at least one exposure or outlying self-reported physical activity values, the analytic sample comprised 350,512 participants (Fig. 1).

### Assessment of physical activity, grip strength and sedentary behaviour

At the baseline visit, intensity-specific physical activity was assessed using a modified version of the International Physical Activity Questionnaire Short Form (IPAQ-SF) using a touchscreen. Frequency per week, and duration per day (minimum of 10 min), of walking, moderate and vigorous physical activities (e.g. at work, at home, for commuting and during leisure time) during the past 4 weeks were recorded. Validation of the IPAQ-SF showed good test-retest reliability (pooled correlation coefficient 0.76, 95% CI 0.73–0.77) and fair agreement with accelerometry (pooled correlation coefficient 0.30, 95% CI 0.23–0.36).^19^ Data processing was conducted as recommended by the IPAQ Scoring Protocol.^20^ First, participants with missing data or implausibly high values (>960 min per day of the sum total of walking, moderate and vigorous physical activity) were excluded from analyses. Next, physical activity durations <10 min per day were recoded as 0 min per day. Durations exceeding 3 h of walking, moderate and vigorous physical activity were recoded to 180 min per day each. The average metabolic equivalent of task (MET) values from the Compendium by Ainsworth et al.^21^ recommended for walking (3.3 METs), moderate (4.0 METs) and vigorous (8.0 METs) physical activities were multiplied with the frequency and duration of those activities to obtain MET-minutes per week.^20^ Intensity-specific MET-minutes were summed to obtain total MET-minutes per week of moderate to vigorous physical activity (MVPA). Grip strength was measured during the baseline examination using the Jamar J00105 hydraulic hand dynamometer (Lafayette Instrument, USA) and the mean of the right and left hand values, in kg, was used in the analysis.^22^ In the touchscreen questionnaire, three specific questions on duration of sedentary behaviours on a typical day (or the average duration for a 24 h day in the last 4 weeks) were asked. The sum of time spent watching TV, using a computer during leisure time and driving for transportation was calculated to obtain total sedentary behaviour per day, as described previously.^5^ If this sum exceeded 24 h per day, total sedentary behaviour was recoded to 24 h per day.

### Cohort follow-up and ascertainment of malignant melanoma cases

Participants’ vital status was ascertained by linkage to routine health care data (UK National Health Service) and national death registries.^18^ A previous study showed that completeness of case ascertainment in cancer registries in England is very high (about 98–99%).^23^ Depending on the diagnosis date, classification of cancer was conducted with the International Classification of Diseases 9th or 10th Revision (ICD-9 or ICD-10).^24,25^ Tumour morphology and histology originated from the ICD-O-3. Date of complete follow-up was March 31, 2016 for England and Wales and October 31, 2015 for Scotland. Malignant melanoma was defined as the combination of ICD-10 code C43 and histology codes 8720–8790.^26^ Malignant melanoma of the lip (C43.0), the eyelid including canthus (C43.1), the ear and external auricular canal (C43.2), other and unspecified parts of the face (C43.3), scalp and neck (C43.4) and upper limb, including shoulder (C43.6), were classified as malignant melanoma at chronically UV-exposed skin areas.^27^ Malignant melanoma of the trunk (C43.5) and of the lower limb, including the hip (C43.7), were classified as malignant melanoma at intermittently UV-exposed skin areas.^27^

### Covariables

We identified de-confounders and backdoor paths a priori with directed acyclic graphs and the disjunctive cause criterion.^28^ Direct causes of the exposure or outcome, excluding instrumental variables, were assumed to represent a confounder set (Fig. S1, Appendix. S1).^28^ Basic models were adjusted for age, gender (women/men) and study region (England, Wales, Scotland). In fully adjusted models, we further included education (University or College degree/A-levels, AS-levels, NVQ, HND, HNC or equivalent or other professional qualification/O-levels, CSEs or equivalent/none of the above), smoking behaviour (pack years), alcohol consumption (g/d),^29^ height (cm), coffee consumption (cups per day), solarium use (yes/no), sunburn occasions during childhood (painful sunburn for at least 2 days or sunburn that caused blistering before the age of 15 years, yes/no), time spent outdoors during the summer (hours per typical day) and seasonality (sine-cosine terms for month of baseline assessment center visit). Models were mutually adjusted for physical activity (MET-minutes per week of MVPA), grip strength (kg) and sedentary behaviour (hours per day). We generated an indicator variable for UV sensitivity using information on the combination of skin tanning (get very tanned/get moderately tanned/get mildly or occasionally tanned/never tan, only burn), skin colour without tanning (black, brown/light or dark olive/fair/very fair) and natural hair colour before turning grey (other, black, dark brown/light brown/blonde, red) (Supplement Table S1 and Fig. S2). Models were either adjusted for or stratified by this UV sensitivity score. In a further analysis in participants who were currently (self-) employed, we additionally adjusted for being employed at a workplace with UV exposure (yes/no). Participant’s occupation was coded with the standard occupational classification system. We used results from previous studies to identify jobs with harmful natural or artificial UV exposures (Appendix Table S2).^30,31^

### Statistical analyses

We report age-adjusted baseline characteristics of the study population with direct standardisation to the age distribution of the cohort, stratified by gender-specific quartiles of physical activity, grip strength and sedentary behaviour. We used inverse probability weighted Kaplan–Meier curves to obtain the weighted number of malignant melanoma cases in each tertile of physical activity, grip strength and sedentary behaviour^32,33^ and estimated adjusted incidence rates and corresponding exact 95% confidence intervals (CI) per 10,000 person-years.^34^ Missing covariate values were multiply imputed using chained-equations (10 datasets with 5 iterations each) in order to increase statistical power.^35^ Predictive mean matching (for continuous variables), logistic regression (for binary variables), ordered logit models (for ordinal variables) and multinomial logit models (for nominal variables) were employed as imputation models, and convergence and plausibility of the imputation approach were assessed using diagnostic plots.^35^ Hazard ratios (HR) with corresponding 95% CI for the relations of physical activity (MET-minutes per week of MVPA), grip strength (kg) and sedentary behaviour (hours per day) to the incidence of malignant melanoma were estimated using Cox proportional hazards regression models. We report HRs per interquartile range increase in the exposure variable (i.e. 25–75th percentile) to ease comparability across exposures.^36^ Additionally, HRs are reported per unit increase (100 MET-minutes, 1 kg in grip strength and 1 h of sedentary behaviour) and for high versus low exposure (cut points for age- and gender-specific tertiles are reported in Table S3) to ease interpretability. Age was chosen as the underlying time metric.^37^ Age in years at baseline was employed as entry time. Age at complete follow-up, at diagnosis of malignant melanoma or at death, whichever came first, was used as exit time. Non-linearity of the exposure-outcome association was checked using restricted cubic splines with four knots at fixed quantiles (0.05, 0.35, 0.65, 0.95) of the distribution for physical activity and grip strength as well as three knots at fixed quantiles (0.10, 0.50, 0.90) of the distribution for sedentary behaviour.^36^ A test of non-linearity was conducted by testing the coefficient of the second spline (three knots) or the second and third spline (four knots) transformation equal to zero. In models testing for non-linearity of the association between physical activity and malignant melanoma, we included a dummy variable (physical activity yes/no) to consider the spike at zero, based on an approach described previously.^38^ If the tests indicated non-linearity, we showed this relationship in a plot using restricted cubic splines. If the proportional hazards assumption, tested with Schoenfeld residuals, was violated, we ran stratified Cox models. Separate models were run for the overall study population and for women and men separately, as well as for total malignant melanoma and anatomic location-specific malignant melanomas (chronically UV-exposed skin versus intermittently UV-exposed skin). Additionally, we ran analyses stratified by UV sensitivity in participants with observed exposure and UV sensitivity data. We performed sensitivity analyses to test the robustness of our assumptions. Specifically, to minimise the potential for reverse causality we excluded malignant melanoma cases that occurred during the first 2 years of follow-up.^39^ Residual confounding by baseline health status was minimised by excluding participants with subjectively rated ”poor“ health. In addition to the multiply imputed models, a complete case model was considered. All analyses were conducted using R Statistical Software version 3.6.1.^40^

## Results

### Descriptive statistics

During 2,472,298 person-years of follow-up (mean = 7 years, standard deviation = 1 year), 1239 incident malignant melanoma cases occurred. Of these, 507 (40.9%) were at locations with chronic UV exposure and 712 (57.5%) were at locations with intermittent UV exposure (20 participants developed malignant melanoma at overlapping or unspecified locations). Participants reported on average 2657.0 (standard deviation (SD) = 2655.0) MET-minutes per week of MVPA (1.8% of participants reported doing no MVPA) and 4.8 (SD = 2.4) sedentary hours per day. Measurement of grip strength yielded on average 31.7 (SD = 11.0) kg. Incidence rates of malignant melanoma were slightly higher in the third tertile of physical activity, grip strength and sedentary behaviour as compared to the first tertile (Table S4).

Participants with high levels of physical activity had higher grip strength and were less sedentary than participants with lower physical activity levels. The greatest proportion of participants reporting excellent health was seen in the highest quartiles of physical activity or grip strength. A graded increase in standing height was noted across increasing quartiles of grip strength. Participants in the highest quartiles of physical activity, grip strength or sedentary behaviour reported more time spent outdoors in the summer (Table 1, Tables S5 and S6).

**Table. 1:** Age-standardised characteristics of the study population at baseline by quartiles of physical activity (self-reported).

	Gender-specific quartile of subjective physical activity (MET-minutes/week of MVPA); *n* = 350,512				
	1st Quartile lowest PA	2nd Quartile	3rd Quartile	4th Quartile highest PA	Missing values
Number of participants	87,705	87,680	87,632	87,495	
Gender					0
Women (%)	50.6	50.9	50.8	51.0	
Age (years)	55.9	56.0	56.0	56.0	0
MET-minutes per week of MVPA	419.0	1286.2	2571.8	6362.2	0
Grip strength (kg)	30.9	31.6	32.0	32.2	0
Sedentary behaviour (h/d)	5.2	4.8	4.6	4.6	0
Region					0
England	88.3	88.3	88.6	89.5	
Wales	4.6	4.0	3.9	3.9	
Scotland	7.1	7.6	7.5	6.6	
Education					1999
University/College	37.3	41.3	39.0	28.1	
A-/AS-levels, NVQ, HND, HNC, equivalent, other professional qualification	23.4	23.2	23.2	24.6	
O-levels, CSEs, equivalent	26.0	24.4	25.6	30.0	
None of the above	13.3	11.1	12.2	17.3	
Smoking (pack years)	7.7	6.1	6.0	6.9	6452
Alcohol (g/d)	17.5	17.5	17.9	18.7	49,841
Coffee (cups/d)	2.1	2.0	2.0	2.0	153
Standing height (cm)	169.3	169.5	169.4	168.9	309
Overall health rating					812
Excellent	12.1	17.5	22.0	22.7	
Good	55.1	60.8	60.3	59.3	
Fair	25.5	18.9	15.5	16.0	
Poor	7.3	2.8	2.2	2.0	
UV sensitivity score category					9757
Low UV sensitivity	42.3	43.0	44.0	46.0	
High UV sensitivity	57.7	57.1	56.0	54.0	
Time spent outdoors summer (h/d)	3.0	3.4	3.8	4.7	7497
Sunburn occasions in childhood	1.7	1.8	1.8	1.7	75,150
Solarium/Sunlamp use (%)	9.0	9.2	9.6	10.2	1675
Season during baseline assessment					0
Spring	28.7	28.6	28.3	28.7	
Summer	24.8	25.6	26.7	28.1	
Autumn	23.7	24.1	24.4	24.1	
Winter	22.8	21.7	20.7	19.1	
Job with UV exposure (%)^a^	4.7	4.9	6.8	16.5	5

Age standardisation was done by direct standardisation to the age distribution of the cohort at baseline.

*PA* physical activity, *MET* metabolic equivalent of task, *UV* ultraviolet radiation.

^a^In a subset of 212,700 participants who are currently in paid employment or self-employed.

### Physical activity

No association was evident between physical activity and total malignant melanoma, malignant melanoma at chronically UV-exposed skin or at intermittently UV-exposed skin as well as in models stratified by gender or UV sensitivity, neither in basic nor in fully adjusted models (see Tables 2 and 3). After full adjustment, restricted cubic splines indicated a non-linear association between physical activity and malignant melanoma at chronically UV-exposed skin in women (see Fig. S3C). The fully adjusted HR of total malignant melanoma per 100 MET-minutes increase was 1.00 (95% CI 1.00–1.00) and the fully adjusted HR of total malignant melanoma for high versus low physical activity was 1.04 (95% CI 0.90–1.20).

**Table. 2:** Association between physical activity, grip strength, sedentary behaviour and malignant melanoma (at chronically/intermittently UV-exposed skin).

	Total malignant melanoma			Malignant melanoma at chronically UV-exposed skin			Malignant melanoma at intermittently UV-exposed skin		
	Cases	HR (95% CI)	*p*-value	Cases	HR (95% CI)	*p*-value	Cases	HR (95% CI)	*p*-value
Physical activity—basic model									
Overall	1239	1.00 (0.95–1.06)	0.892	507	0.95 (0.86–1.04)	0.260	712	1.05 (0.97–1.13)	0.211
Women	559	1.03 (0.94–1.12)	0.519	211	1.03 (0.90–1.19)	0.666	339	1.04 (0.93–1.16)	0.465
Men	680	0.99 (0.91–1.07)	0.749	296	0.90 (0.79–1.02)	0.101	373	1.05 (0.95–1.16)	0.331
Physical activity—full model									
Overall	1239	1.01 (0.95–1.07)	0.779	507	0.98 (0.89–1.08)	0.675	712	1.03 (0.95–1.12)	0.440
Women	559	1.03 (0.94–1.13)	0.554	211	1.04 (0.90–1.20)	0.606^a^	339	1.03 (0.92–1.16)	0.573
Men	680	1.00 (0.92–1.09)	0.958	296	0.94 (0.82–1.08)	0.382	373	1.03 (0.92–1.15)	0.591
Grip strength—basic model									
Overall	1239	1.23 (1.08–1.40)	0.002^b^	507	0.99 (0.81–1.22)	0.958	712	1.39 (1.17–1.66)	<0.001
Women	559	1.08 (0.96–1.21)	0.178	211	0.93 (0.77–1.12)	0.417	339	1.17 (1.01–1.36)	0.036
Men	680	1.16 (1.04–1.29)	0.006	296	1.04 (0.89–1.22)	0.637	373	1.25 (1.08–1.44)	0.002
Grip strength—full model									
Overall	1239	1.10 (0.96–1.26)	0.178	507	0.92 (0.74–1.13)	0.425	712	1.22 (1.02–1.47)	0.031
Women	559	1.01 (0.89–1.14)	0.900	211	0.87 (0.72–1.06)	0.179	339	1.08 (0.92–1.26)	0.359
Men	680	1.10 (0.98–1.22)	0.101	296	0.99 (0.84–1.17)	0.929	373	1.17 (1.01–1.36)	0.035
Sedentary behaviour—basic model									
Overall	1239	1.02 (0.95–1.09)	0.657	507	0.95 (0.85–1.07)	0.404	712	1.06 (0.97–1.16)	0.231^a^
Women	559	1.04 (0.95–1.15)	0.404	211	0.96 (0.82–1.13)	0.638	339	1.09 (0.97–1.23)	0.143^a^
Men	680	1.00 (0.91–1.09)	0.951	296	0.96 (0.83–1.10)	0.530	373	1.02 (0.91–1.15)	0.740
Sedentary behaviour—full model									
Overall	1239	1.04 (0.97–1.12)	0.306	507	0.99 (0.88–1.11)	0.835	712	1.07 (0.97–1.18)	0.164^a^
Women	559	1.07 (0.97–1.19)	0.161	211	1.00 (0.84–1.18)	0.963	339	1.12 (0.99–1.27)	0.067^a^
Men	680	1.01 (0.92–1.11)	0.755	296	0.98 (0.85–1.14)	0.832	373	1.03 (0.91–1.16)	0.686

Cox proportional hazards regression models with observed exposure and multiply imputed covariable data (overall *n* = 350,512; women *n* = 178,340; men *n* = 172,172). Exposures (physical activity, grip strength, sedentary behaviour) were entered as continuous variables. Associations were modelled linearly and hazard ratios are shown for an interquartile range increase (i.e. 25–75th percentile):

Physical activity: increment of 2751/2627.5/2910 MET-minutes per week of MVPA (overall/women/men).

Grip strength: increment of 17/8/11.5 kg (overall/women/men).

Sedentary behaviour: increment of 3/2.5/3 h per day (overall/women/men).

Basic models were adjusted for age (as time scale), study region (baseline hazard stratification) and in the overall group additionally for gender.

Full models were additionally adjusted for education, pack years, alcohol consumption, coffee consumption, height, UV sensitivity, time spent outdoors during summer, sunburn occasions during childhood, solarium use and seasonality. Models were also mutually adjusted for physical activity, grip strength and sedentary behaviour.

^a^Indication for non-linear association. Restricted cubic spline plots in the supplement (Fig. S3).

^b^Potential violation of the proportional hazards assumption regarding grip strength.

**Table. 3:** Association between physical activity, grip strength, sedentary behaviour and malignant melanoma stratified by UV sensitivity.

	Low UV sensitivity^a^			High UV sensitivity^b^		
	Cases	HR (95% CI)	*p*-value	Cases	HR (95% CI)	*p*-value
Physical activity						
Overall	372	0.98 (0.87–1.10)	0.755	846	1.02 (0.95–1.10)	0.574
Women	140	1.00 (0.83–1.20)	0.983	412	1.03 (0.93–1.14)	0.613
Men	232	0.98 (0.84–1.13)	0.751	434	1.02 (0.92–1.13)	0.711
Grip strength						
Overall	372	1.29 (1.01–1.64)	0.043	846	1.03 (0.88–1.20)	0.743
Women	140	1.07 (0.84–1.37)	0.559	412	0.99 (0.86–1.14)	0.862
Men	232	1.22 (1.00–1.48)	0.050	434	1.05 (0.91–1.21)	0.488
Sedentary behaviour						
Overall	372	1.09 (0.96–1.24)	0.178	846	1.00 (0.92–1.10)	0.935
Women	140	1.11 (0.92–1.35)	0.264	412	1.04 (0.93–1.17)	0.500
Men	232	1.07 (0.92–1.25)	0.403	434	0.98 (0.87–1.10)	0.747

Cox proportional hazards regression models with observed exposure and UV sensitivity and multiply imputed covariable data. Exposures (physical activity, grip strength, sedentary behaviour) were entered as continuous variables. Associations were modelled linearly and hazard ratios are shown for an interquartile range increase (i.e. 25–75th percentile):

^a^Low UV sensitivity: UV sensitivity score < 4 (overall *n* = 149,261; women *n* = 66,721; men *n* = 82,540).

Physical activity: increment of 2877/2720/2988 MET-minutes per week of MVPA (overall/women/men).

Grip strength: increment of 17/8/12 kg (overall/women/men).

Sedentary behaviour: increment of 3/2.5/3 h per day (overall/women/men).

^b^High UV sensitivity: UV sensitivity score ≥ 4 (overall *n* = 191,494; women *n* = 106,909; men *n* = 84,585).

Physical activity: increment of 2656/2532/2791.5 MET-minutes per week of MVPA (overall/women/men).

Grip strength: increment of 16/8/11.5 kg (overall/women/men).

Sedentary behaviour: increment of 3/2.5/3 h per day (overall/women/men).

Models were adjusted for age (as time scale), study region (baseline hazard stratification), gender (only in the overall group), education, pack years, alcohol consumption, coffee consumption, height, time spent outdoors during summer, sunburn occasions during childhood, solarium use and seasonality. Models were also mutually adjusted for physical activity, grip strength and sedentary behaviour.

Results were comparable in the complete case model with 222,000 participants and 776 cases of incident malignant melanoma (see Table S7), as well as after additional adjustment for UV exposure at work in a subgroup of 212,700 participants (accruing 655 incident malignant melanoma cases) currently (self-) employed. Exclusion of 308 cases that occurred within the first 2 years after baseline or exclusion of 13,353 participants (reducing the analysis to 1196 malignant melanoma cases) who reported poor health at study baseline or did not answer that question did not alter the associations materially (data not shown).

### Grip strength

In the basic model, the HR of malignant melanoma per interquartile range increase in grip strength was 1.23 (95% CI 1.08–1.40) (Table 2). The addition of height to the basic model attenuated the HR to 1.09 (95% CI 0.95–1.25). In contrast, adding UV exposure- and sensitivity-related variables to the basic model slightly strengthened the HR (1.24, 95% CI 1.09–1.42). In basic models stratified by gender, the positive association of grip strength and malignant melanoma was apparent in men (HR 1.16, 95% CI 1.04–1.29) but not women (Table 2), although the test for interaction was not statistically significant (p-value for interaction by gender = 0.710). After full adjustment, all confidence intervals included the null value of 1.0. The fully adjusted HR of total malignant melanoma per 1-kg-increase in grip strength was 1.01 (95% CI 1.00–1.01) and the fully adjusted HR of total malignant melanoma for high versus low grip strength was 1.10 (95% CI 0.95–1.27). No association was evident between grip strength and malignant melanoma at chronically UV-exposed skin. However, grip strength was positively associated with risk of melanoma at intermittently UV-exposed locations in the overall cohort (HR 1.39, 95% CI 1.17–1.66), in men (HR 1.25, 95% CI 1.08–1.44) and in women (HR 1.17, 95% CI 1.01–1.36) in the basic models. After full adjustment, positive associations remained evident in the overall group and in men (Table 2). In models stratified by UV sensitivity (Table 3), grip strength was positively associated with risk of melanoma in participants with low UV sensitivity (HR 1.29, 95% CI 1.01–1.64).

In the fully adjusted complete case analysis, all confidence intervals for the associations between grip strength and risk of total malignant melanoma contained the null value of 1.0 (Table S7). In the sample of currently (self-) employed participants, further adjustment for UV exposure at work strengthened the associations between grip strength and total malignant melanoma in the overall group (HR 1.20, 95% CI 1.00–1.44) and in women (HR 1.15, 95% CI 0.97–1.37). Exclusion of 308 cases that occurred within the first 2 years after baseline strengthened the associations between grip strength and total malignant melanoma in the overall group (HR 1.17, 95% CI 1.00–1.37) and in men (HR 1.15, 95% CI 1.02–1.31). Exclusion of participants who reported poor health or did not respond to that question did not alter the results materially.

### Sedentary behaviour

All confidence intervals for the basic and fully adjusted associations between sedentary behaviour and total malignant melanoma, malignant melanoma at chronically UV-exposed skin or at intermittently UV-exposed skin as well as in models stratified by gender or UV sensitivity included the null value of 1.0 (Tables 2 and 3). In women and in the overall group, restricted cubic splines indicated a non-linear association between sedentary behaviour and malignant melanoma at intermittently UV-exposed skin (Fig. S3 A, B, D, E). The fully adjusted HR of total malignant melanoma per 1-h-increase in sedentary behaviour was 1.01 (95% CI 0.99–1.04) and the fully adjusted HR of total malignant melanoma for high versus low sedentary behaviour was 1.10 (95% CI 0.96–1.27).

Estimates were materially unaltered in the complete case model (see Table S7) as well as after additional adjustment for UV exposure at work, exclusion of 308 cases that occurred within the first 2 years after baseline, or exclusion of participants who reported poor health at study baseline, or did not answer that question (data not shown).

## Discussion

In this prospective investigation of more than 350,500 women and men followed for up to 9 years, we found no relations of physical activity or sedentary behaviour to risk of malignant melanoma. The initially observed positive relation between grip strength and malignant melanoma in the basic model is most likely attributable to confounding by height.

A recent UK Biobank analysis assessed the association between predicted physical activity energy expenditure and malignant melanoma incidence and found a HR of 1.05 (95% CI 0.93–1.19) in a model adjusted for numerous variables but not for malignant melanoma-specific risk factors.^41^ A previous meta-analysis that investigated the relation of physical activity to risk of malignant melanoma included three cohort studies and five case-control studies.^7^ The case-control studies were mostly adjusted for individual-level UV exposure and showed an inverse but statistically non-significant association (HR 0.85, 95% CI 0.63–1.14). In contrast, the cohort studies, mostly conducted in North America, yielded a HR of 1.27 (95% CI 1.16–1.40) for high versus low physical activity and melanoma.^7^ In our prospective analysis, we did not find a positive association between physical activity and malignant melanoma. Possibly, the relation between physical activity and UV-related skin damage is weaker in Great Britain, where very high UV exposure levels (UV Index = 8) are rare, whereas in North America extreme UV exposure levels (UV index values ≥ 11) can occur.^42,43^ In agreement with our study, a previous pooled study showed no association between physical activity and malignant melanoma in geographic regions with low ground-level solar UV radiation.^26^ Also, outdoor physical activity has been found to be positively associated with ambient temperature and inversely associated with precipitation, possibly favouring indoor sports in Great Britain.^44^ Furthermore, UK Biobank participants were asked about the hours spent outdoors on a typical day in the summer which might result in insufficient discrimination and may possibly reflect chronic but not intermittent UV exposure (e.g. sun bathing during summer holidays). Intermittent exposure to UV radiation has been shown to be a stronger risk factor for malignant melanoma than chronic exposure, possibly due to protective skin adaptions (e.g. tanning and skin thickening) after continuous UV exposure.^11,45,46^

To the best of our knowledge, the current study is the first on grip strength and risk of malignant melanoma. A previous study on cardiorespiratory fitness, another component of physical fitness,^4^ and incident melanoma reported a positive but statistically non-significant association.^47^ In our analyses, the initially positive association between grip strength and malignant melanoma was attenuated after adjustment for multiple individual-level risk factors, especially height. Adult attained height has been found to be a proxy for genetic, environmental, nutritional and hormonal factors, which increase the risk of malignant melanoma.^12,48^ Also, previous studies showed that height is positively associated with grip strength.^49–51^ Height is thus a strong confounder of the association between grip strength and risk of malignant melanoma. Like height, birth weight has been shown to be positively associated with grip strength^52^ and positive associations were found between birth weight and risk of malignant melanoma.^12,53^ While our results might be influenced by confounding, a previous analysis showed that the impact of birth weight was negligible after adjustment for height.^53^ In some of our additional sub-analyses, the positive association between grip strength and melanoma risk remained apparent after full adjustment. As those analyses were also adjusted for physical activity and sedentary behaviour, the positive association might be due to the non-modifiable component of grip strength. Further study, e.g. using Mendelian Randomisation, is needed to provide additional information regarding the association between grip strength and risk of malignant melanoma.

The major strength of the present study is its prospective design with simultaneous control for individual-level data on melanoma-specific influences such as UV exposure or adult attained height. The broad scope of the UK Biobank allowed us to conduct analyses stratified by UV sensitivity and melanoma anatomic site. Grip strength was assessed in an objective fashion, which reduces misclassification bias. Incident malignant melanoma was identified via linkage to national cancer registries, which leads to complete case ascertainment.

Nonetheless, our study also has some limitations. Physical activity and sedentary behaviour were assessed subjectively, which is susceptible to social desirability reporting or recall bias.^54^ The same applies to covariables regarding UV exposure or UV sensitivity. We assumed that such misclassification was non-differential as the study used a prospective design. Also, no quantitative information on occupational sedentary behaviour was available. Longer follow-up time and a larger number of cases would have increased statistical precision, especially in sub-analyses. A further limitation is the use of single exposure assessments which might favor regression dilution bias.^39^ As the latency periods between physical activity, grip strength or sedentary behaviour and initiation of malignant melanoma most likely span several decades, we had to assume that exposure and confounder information at study baseline represent long-term conditions. Reassuringly, a previous meta-analysis showed that UV exposure is similar over time.^11^ The UK Biobank had a low response proportion (5.5 %), which might limit the generalisability of the results. However, a recent study found that UK Biobank risk factor associations can, on the whole, be transferred to the general population.^55^

## Conclusion

Our findings suggest that physical activity, muscular fitness and sedentary behaviour are not associated with risk of malignant melanoma. The positive association between grip strength and malignant melanoma in the basic models vanished after statistical control for height, which emphasises the relevance of adequate control for objectively measured confounding variables at the individual level.

## Supplementary information

Supplementary information

Completed STROBE Checklist

## Data Availability

UK Biobank is an open access resource. Bona fide researchers can apply to use the UK Biobank data set by registering and applying at http://www.ukbiobank.ac.uk/register-apply.
