# Supplementary material for: Association between physical activity, grip strength and sedentary behaviour with incidence of malignant melanoma: results from the UK Biobank
Source: Br J Cancer. 2021 May 31;125(4):593–600. doi: 10.1038/s41416-021-01443-5 (PMC8368160; doi:10.1038/s41416-021-01443-5)
Supplement: Supplementary file 1 — Supplementary information [file 41416_2021_1443_MOESM1_ESM.pdf]

Supplementary information

Article title: Association between physical activity, grip strength, and sedentary behaviour with incidence of malignant melanoma: results from the UK Biobank

Journal: British Journal of Cancer

Authors: Andrea Weber, Michael F. Leitzmann, Anja M. Sedlmeier, Hansjörg Baurecht, Carmen Jochem, Sebastian Haferkamp, Sebastian E. Baumeister

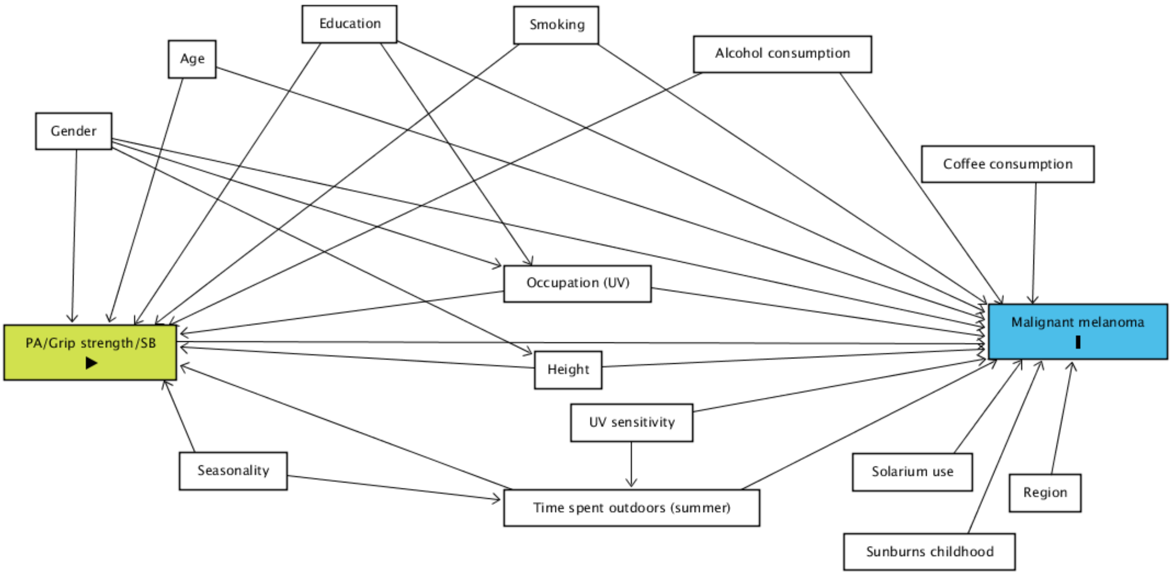

Figure S1: Directed acyclic graph (DAG) representing causal and confounding paths between the exposure (physical activity, grip strength and sedentary behaviour) and malignant melanoma.[1]

Appendix S1: Covariable selection

A study showed that higher education levels were associated with an increased risk of malignant melanoma.[2] Also, people employed in e.g., agriculture, military service, construction or transport as well as some metal forming, welding or instrument making trades have an exposure to natural or artificial UV radiation at work.[3] The effect of smoking on the risk of malignant melanoma is unclear, however cigarette smoke contains more than 60 carcinogens.[4] There is limited suggestive evidence for a beneficial effect of coffee on malignant melanoma in women and for a harmful effect of alcohol consumption on malignant melanoma.[5,6] Adult attained height has been found to be a proxy for genetic, environmental, nutritional and hormonal factors which increase the risk of malignant melanoma.[5,7] According to previous studies, height is also associated with grip strength.[8-10] Physical activity, sedentary behaviour and fitness are associated with time spent outdoors.[11] As participants were asked about their physical activity of the previous 4 weeks, and recall about time spent outdoors in summer might differ by baseline examination date, seasonality could influence these variables. Solarium usage as well as sunburn during childhood are also risk factors for malignant melanoma.[12-15] We stratified baseline hazards according to region as it might act as a proxy for ground-level solar UV radiation. We did not adjust for ethnicity as we had individual level information on skin colour, hair colour and ease of skin tanning, which is associated with ethnicity but more informative in relation to sun sensitivity. We did also not adjust for the use of sun/UV protection (e.g., sunscreen lotion, wearing

a hat when outdoors in the summer) because we assumed the measurement bias affecting that variable would increase bias rather than decrease it.[16] In previous studies, sunscreen use during intermittent sun exposure was shown to be associated with a higher risk of melanoma, possibly due to the prolonged time spent in the sun and thus increased exposure to harmful radiation.[16] In contrast to sunscreen usage, protective clothing was shown to be associated with a decreased risk of melanoma, however, UK Biobank did not distinguish between sunscreen use and protective clothing.[17] As no association was found between genetically determined BMI and malignant melanoma, we did not adjust for measures of body composition.[7] Concerns about UV related skin damage have been shown to act as a barrier to outdoor activities.[18] Also, people with red hair, light skin, freckles, light eyes, sun sensitivity and little skin tan have an approximately 50% higher risk of malignant melanoma than people with no such characteristics.[19] This indicates that UV exposure has a different effect on people with higher UV sensitivity. Therefore, we ran additional analyses stratified by UV sensitivity. UK Biobank participants provided information on their skin colour (without tanning), their natural hair colour (before greying) and the reaction of their skin at repeated exposure to bright sunlight without any protection (ease of skin tanning). We summed these items and weighted individual responses according to a weighting scheme (Supplementary Table S1) to create a UV sensitivity score ranging from 0 to 8 with higher scores indicating greater UV sensitivity. We established the face validity of our UV sensitivity score by examining average number of sunburns in childhood and number of malignant melanoma cases per 10,000 person-years in each stratum of UV sensitivity (Supplementary Figure S2).

Table S1: Weighing of answer possibilities for the creation of the UV sensitivity score.

**Skin colour (without tanning)**	
Brown, black	0
Light, dark olive	1
Fair	2
Very fair	3
**Natural hair colour (before grey)**	
Black, dark brown, other	0
Light brown	1
Blonde, red	2
**Ease of skin tanning**	
Get very tanned	0
Get moderately tanned	1
Get mildly or occassionally tanned	2
Never tan, only burn	3

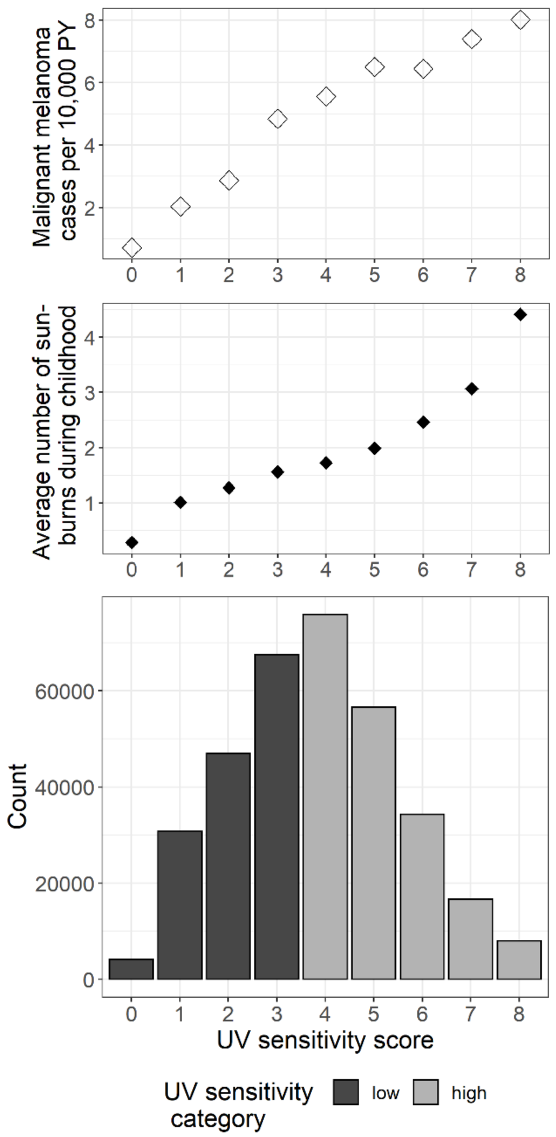

Figure S2: Distribution of UV sensitivity and face validity of the UV sensitivity score.

Table S2: List of occupations with natural or artificial UV exposure.

SOC-code	Occupation
	**Construction**
5214	Metal plate workers, shipwrights, riveters
5311	Steel erectors
5312	Bricklayers, masons
5313	Roofers, roof tilers and slaters
5315	Carpenters and joiners
5316	Glaziers, window fabricators and fitters
5319	Construction trades n.e.c.
5321	Plasterers
5322	Floorers and wall tilers
5323	Painters and decorators
8141	Scaffolders, stagers, riggers
8142	Road construction operatives
8143	Rail construction and maintenance operatives
8149	Construction operatives n.e.c.
8221	Crane drivers
8229	Mobile machine drivers and operatives n.e.c.
9121	Labourers in building and woodworking trades
9129	Labourers in other construction trades n.e.c.
	**Agriculture/ Horticulture/Forestry**
5111	Farmers
5112	Horticultural trades
5113	Gardeners and groundsmen/groundswomen
5119	Agricultural and fishing trades n.e.c.
8223	Agricultural machinery drivers
9111	Farm workers
9112	Forestry workers
9119	Fishing and agriculture related occupations n.e.c.
	**Military service/ Police/ Fire service**
3311	NCOs and other ranks
3312	Police officers (sergeant and below)
3313	Fire service officers (leading fire officer and below)

SOC-code	Occupation
	**Transport**
3513	Ship and hovercraft officers
8211	Heavy goods vehicle drivers
8212	Van drivers
8213	Bus and coach drivers
8214	Taxi, cab drivers and chauffeurs
8215	Driving instructors
8216	Rail transport operatives
8217	Seafarers (merchant navy)
8218	Air transport operatives
8219	Transport operatives n.e.c.
	**Other**
3449	Sports and fitness occupations n.e.c.
3552	Countryside and park rangers
5243	Lines repairers and cable jointers
6139	Animal care occupations n.e.c.
6211	Sports and leisure assistants
7124	Market and street traders and assistants
8123	Quarry workers and related operatives
8222	Fork-lift truck drivers
9211	Postal workers, mail sorters, messengers, couriers
9226	Leisure and theme park attendants
9231	Window cleaners
9232	Road sweepers
9235	Refuse and salvage occupations
	**Artificial UV exposure**
5211	Smiths and forge workers
5213	Sheet metal workers
5215	Welding trades
5221	Metal machining setters and setter-operators
5222	Tool makers, tool fitters and markers-out
5223	Metal working production and maintenance fitters
5314	Plumbers, heating and ventilating engineers
8132	Assemblers (vehicles and metal goods)

SOC: Standard occupational classification.

Table S3: Cut points for age- and gender-specific tertiles of physical activity, grip strength and sedentary behaviour

	Gender and age (years) groups					
	Women			Men		
	≤ 50	> 50 & ≤ 60	> 60	≤ 50	> 50 & ≤ 60	> 60
**Cut points for physical activity categories (tertiles)**						
**33.3%**	1057.5	1017.0	1253.0	1146.5	1038.0	1199.5
**66.6%**	2570.0	2580.0	3045.0	2895.0	2731.0	3066.0
**Cut points for grip strength categories (tertiles)**						
**33.3%**	24.0	21.0	19.0	39.0	37.0	34.0
**66.6%**	29.0	26.0	24.0	47.0	44.0	40.5
**Cut points for sedentary behaviour categories (tertiles)**						
**33.3%**	3.0	3.5	3.5	4.0	4.0	4.0
**66.6%**	4.5	5.0	5.0	6.0	6.0	6.0

Table S4: Incidence rates of total malignant melanoma by exposure

	Tertile of exposure		
	1st tertile lowest	2nd tertile	3rd tertile highest
**Incidence rate (95 % CI) per 10,000 person years**			
**Physical activity**	4.4 (3.8-5.0)	5.1 (4.5-5.8)	5.4 (4.8-6.1)
**Grip strength**	4.7 (4.1-5.4)	4.3 (3.7-4.9)	5.8 (5.2-6.5)
**Sedentary behaviour**	4.7 (4.1-5.4)	5.2 (4.6-5.7)	4.9 (4.3-5.6)

Incidence rates were adjusted for age, gender, study region, education, pack years, alcohol consumption, coffee consumption, height, UV sensitivity, time spent outdoors during summer, sunburn occasions during childhood, solarium use and seasonality and mutually for physical activity, grip strength and sedentary behaviour.

Table S5: Age-standardized characteristics of the study population at baseline by quartiles of grip strength.

	Gender-specific quartile of grip strength (kg); n= 350,512				
	1st Quartile lowest grip s.	2nd Quartile	3rd Quartile	4th Quartile highest grip s.	Missing values
**Number of participants**	96,289	91,035	79,557	83,631	
**Gender**					0
Women (%)	53.8	48.8	51.9	46.6	
**Age (years)**	56.3	56.1	55.9	55.5	0
**MET-minutes per week of MVPA**	2448.8	2642.1	2722.3	2821.7	0
**Grip strength (kg)**	22.4	30.2	34.3	41.8	0
**Sedentary behaviour (h/d)**	4.9	4.8	4.8	4.8	0
**Region**					0
England	91.3	89.7	87.9	85.1	
Wales	3.8	4.0	4.3	4.3	
Scotland	4.9	6.3	7.8	10.7	
**Education**					1999
University/College	33.3	36.1	38.1	39.8	
A-/AS-levels, NVQ, HND, HNC, equivalent, other professional qualification	22.9	23.6	24.0	24.6	
O-levels, CSEs, equivalent	27.3	27.0	26.0	25.0	
None of the above	16.6	13.3	11.9	10.7	
**Smoking (pack years)**	6.9	6.7	6.5	6.6	6452
**Alcohol (g/d)**	16.1	18.0	18.3	19.5	49,841
**Coffee (cups/d)**	2.0	2.0	2.0	2.1	153
**Standing height (cm)**	166.6	168.9	169.8	172.5	309
**Overall health rating**					812
Excellent	13.7	18.1	20.6	22.7	
Good	55.3	59.8	60.6	60.1	
Fair	24.2	18.9	16.5	15.2	
Poor	6.9	3.2	2.3	1.9	
**UV sensitivity score category**					9757
Low UV sensitivity	43.1	43.6	43.7	45.3	
High UV sensitivity	57.0	56.4	56.3	54.7	
**Time spent outdoors summer (h/d)**	3.6	3.7	3.8	3.9	7497
**Sunburn occasions in childhood**	1.6	1.7	1.8	2.0	75,150
**Solarium/Sunlamp use (%)**	9.1	9.4	9.7	9.8	1675
**Season during baseline assessment**					0
Spring	30.1	28.8	28.1	27.0	
Summer	27.7	26.7	25.9	24.4	
Autumn	22.6	23.7	24.7	25.5	
Winter	19.6	20.8	21.3	23.2	
**Job with UV exposure (%)**[a]	7.0	7.4	8.4	10.0	5

Age standardization was done by direct standardization to the age distribution of the cohort at baseline. MET = Metabolic equivalent of task, UV = Ultraviolet radiation.
[a] In a subset of 212,700 participants who are currently in paid employment or self-employed.

Table S6: Age-standardized characteristics of the study population at baseline by quartiles of sedentary behaviour (self-reported).

	Gender-specific quartile of subjective sedentary behaviour (hours/day); n= 350,512				
	1st Quartile lowest SB	2nd Quartile	3rd Quartile	4th Quartile highest SB	Missing values
**Number of participants**	106,899	93,201	70,949	79,463	
**Gender**					
Women (%)	55.0	43.0	57.4	48.9	0
**Age (years)**	55.9	56.0	56.1	56.0	0
**MET-minutes per week of MVPA**	2793.3	2760.2	2581.4	2430.7	0
**Grip strength (kg)**	31.1	33.1	30.7	31.6	0
**Sedentary behaviour (h/d)**	2.5	4.2	5.4	8.2	0
**Region**					0
England	88.0	88.5	89.1	89.6	
Wales	3.8	4.3	4.2	4.1	
Scotland	8.2	7.2	6.8	6.4	
**Education**					1999
University/College	46.4	36.2	32.1	27.3	
A-/AS-levels, NVQ, HND, HNC, equivalent, other professional qualification	22.0	24.4	24.3	24.3	
O-levels, CSEs, equivalent	21.5	26.4	30.0	30.2	
None of the above	10.1	13.0	13.6	18.2	
**Smoking (pack years)**	4.9	6.4	6.8	9.2	6452
**Alcohol (g/d)**	16.5	19.0	17.6	18.8	49,841
**Coffee (cups/d)**	1.9	2.0	2.1	2.1	153
**Standing height (cm)**	169.0	170.3	168.3	169.1	309
**Overall health rating**					812
Excellent	23.6	18.4	16.8	13.5	
Good	59.3	60.4	60.4	55.4	
Fair	14.8	18.4	19.5	24.9	
Poor	2.4	2.9	3.3	6.3	
**UV sensitivity score category**					9757
Low UV sensitivity	43.0	44.3	42.8	45.2	
High UV sensitivity	57.0	55.7	57.2	54.8	
**Time spent outdoors summer (h/d)**	3.4	3.7	3.8	4.2	7497
**Sunburn occasions in childhood**	1.8	1.8	1.7	1.8	75,150
**Solarium/Sunlamp use (%)**	8.8	9.2	10.2	10.3	1675
**Season during baseline assessment**					0
Spring	27.8	28.5	29.1	29.4	
Summer	27.9	26.4	25.3	24.9	
Autumn	24.3	24.1	24.2	23.5	
Winter	20.0	21.0	21.5	22.1	
**Job with UV exposure (%)**[a]	4.8	7.8	7.5	14.1	5

Age standardization was done by direct standardization to the age distribution of the cohort at baseline. SB = sedentary behaviour, MET = Metabolic equivalent of task, UV = Ultraviolet radiation.

[a] In a subset of 212,700 participants who are currently in paid employment or self-employed.

Table S7: Complete case analysis: association between physical activity, grip strength, sedentary behaviour and malignant melanoma (at chronically/intermittently UV exposed skin).

	Total malignant melanoma			Malignant melanoma at chronically UV exposed skin			Malignant melanoma at intermittently UV exposed skin		
	**Cases**	**HR (95 % CI)**	**p-value**	**Cases**	**HR (95 % CI)**	**p-value**	**Cases**	**HR (95 % CI)**	**p-value**
**Physical activity**									
Overall	776	1.06 (0.99-1.15)	0.096	314	1.04 (0.92-1.17)	0.571	445	1.09 (0.99-1.19)	0.082
Women	346	1.09 (0.97-1.21)	0.147	134	1.09 (0.91-1.30)	0.369	205	1.11 (0.96-1.27)	0.159
Men	430	1.05 (0.95-1.17)	0.328	180	1.00 (0.85-1.19)	0.957	240	1.08 (0.95-1.23)	0.242
**Grip strength**									
Overall	776	1.15 (0.97-1.37)	0.098	314	1.08 (0.83-1.40)	0.586	445	1.17 (0.93-1.46)	0.170
Women	346	1.00 (0.85-1.17)	0.978	134	0.92 (0.72-1.18)	0.522	205	1.03 (0.84-1.26)	0.780
Men	430	1.15 (1.00-1.33)	0.044	180	1.13 (0.91-1.40)	0.266	240	1.16 (0.96-1.40)	0.121
**Sedentary behaviour**									
Overall	776	1.02 (0.93-1.12)	0.674	314	1.04 (0.89-1.20)	0.640	445	1.01 (0.89-1.14)	0.887
Women	346	1.08 (0.95-1.22)	0.256	134	1.15 (0.94-1.40)	0.177	205	1.04 (0.88-1.23)	0.664
Men	430	0.98 (0.87-1.11)	0.771	180	0.96 (0.80-1.17)	0.705	240	0.99 (0.85-1.16)	0.929

Cox proportional hazards regression models with observed exposure and covariable data (complete case analysis; overall n = 222,000; women n = 111,444; men n = 110,556). Exposures (physical activity, grip strength, sedentary behaviour) were entered as continuous variables. Associations were modelled linearly and hazard ratios are shown for an interquartile range increase (i.e., 25th to 75th percentile):
Physical activity: increment of 2735/2637/2883.2 MET-minutes per week of MVPA (overall/women/men)
Grip strength: increment of 16.5/8/11.5 kg (overall/women/men)
Sedentary behaviour: increment of 3/2.5/3 hours per day (overall/women/men)

Fully adjusted models were adjusted for age (as time scale), study region (baseline hazard stratification), gender (in overall group), education, pack years, alcohol consumption, coffee consumption, height, UV sensitivity, time spent outdoors during summer, sunburn occasions during childhood, solarium use and seasonality. Models were also mutually adjusted for physical activity, grip strength and sedentary behaviour.

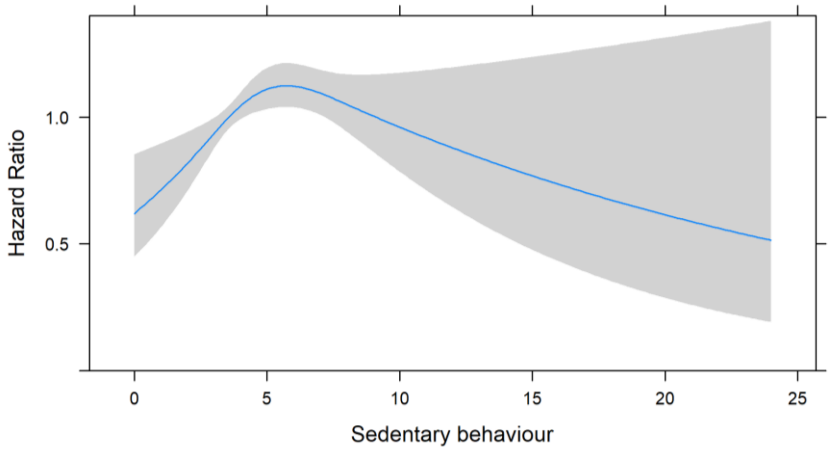

A, Basic model: sedentary behaviour and risk of malignant melanoma at intermittently UV exposed skin in the overall group (n = 350,512).

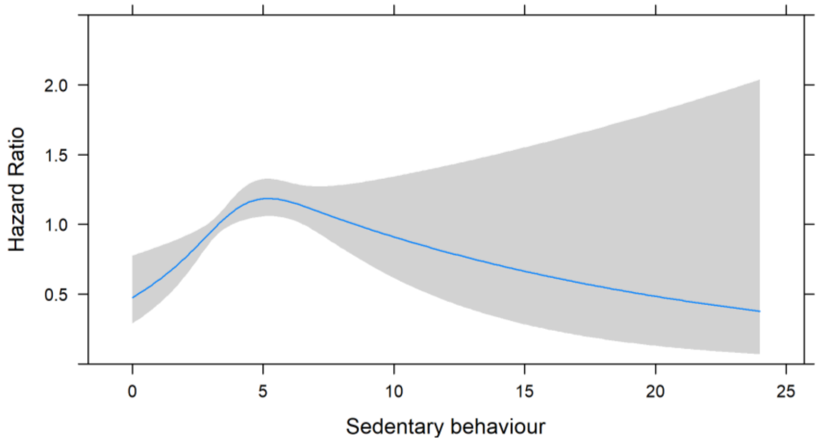

B, Basic model: sedentary behaviour and risk of malignant melanoma at intermittently UV exposed skin in women (n = 178,340).

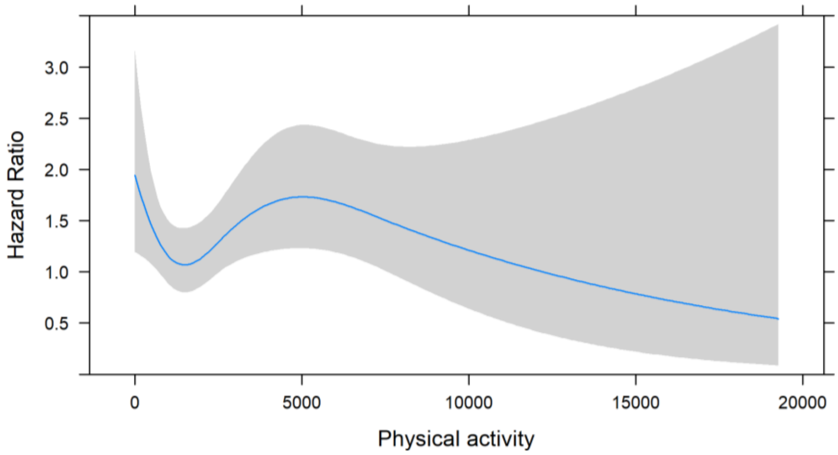

C, Full model: physical activity and risk of malignant melanoma at chronically UV exposed skin in women (n = 178,340).

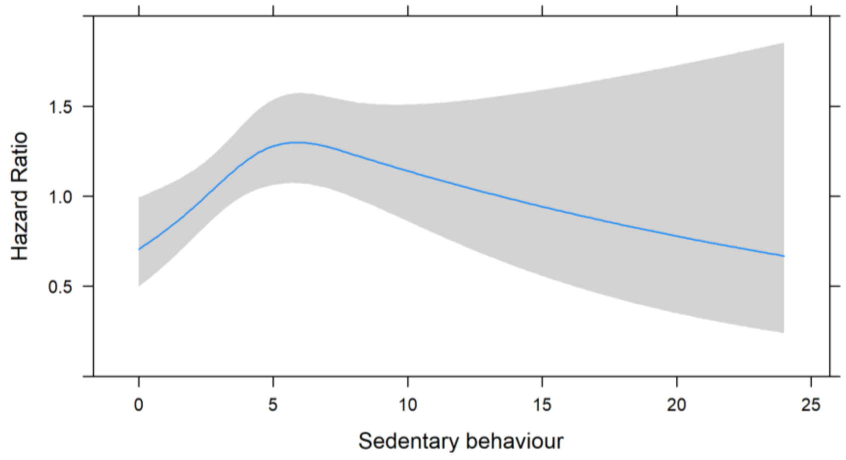

D, Full model: sedentary behaviour and risk of malignant melanoma at intermittently UV exposed skin in the overall group (n = 350,512).

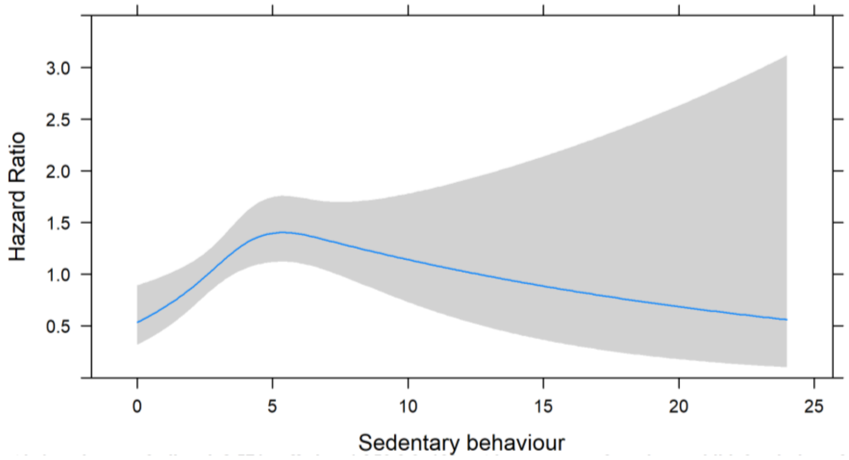

E, Full model: sedentary behaviour and risk of malignant melanoma at intermittently UV exposed skin in women (n = 178,340).

Figure S3: Nonlinear associations using restricted cubic splines.

References Supplementary Information

1 Textor, J., van der Zander, B., Gilthorpe, M. S., Liśkiewicz, M. & Ellison, G. T. H. Robust causal inference using directed acyclic graphs: the R package 'dagitty'. *International Journal of Epidemiology* **45**, 1887-1894 (2016).

2 Lee, P. Y., Silverman, M. K., Rigel, D. S., Vossaert, K. A., Kopf, A. W., Bart, R. S. *et al.* Level of education and the risk of malignant melanoma. *J Am Acad Dermatol* **26**, 59-63 (1992).

3 Surdu, S., Fitzgerald, E. F., Bloom, M. S., Boscoe, F. P., Carpenter, D. O., Haase, R. F. *et al.* Occupational exposure to ultraviolet radiation and risk of non-melanoma skin cancer in a multinational European study. *PloS one* **8**, e62359 (2013).

4 Centers for Disease, C., Prevention, National Center for Chronic Disease, P., Health, P., Office on, S. & Health. Publications and Reports of the Surgeon General. In: *How Tobacco Smoke Causes Disease: The Biology and Behavioral Basis for Smoking-Attributable Disease: A Report of the Surgeon General* (Centers for Disease Control and Prevention (US): Atlanta (GA), 2010).

5 World Cancer Research Fund/ American Insitute for Cancer Research. Continuous Update Project Expert Report 2018. Diet, nutrition, physical activity and skin cancer. Available at dietandcancerreport.org.

6 Micek, A., Godos, J., Lafranconi, A., Marranzano, M. & Pajak, A. Caffeinated and decaffeinated coffee consumption and melanoma risk: a dose-response meta-analysis of prospective cohort studies. *Int J Food Sci Nutr* **69**, 417-426 (2018).

7 Dusingize, J. C., Olsen, C. M., An, J., Pandeya, N., Law, M. H., Thompson, B. S. *et al.* Body mass index and height and risk of cutaneous melanoma: Mendelian randomization analyses. *Int J Epidemiol* 10.1093/ije/dyaa009 (2020).

8 Chandrasekaran, B., Ghosh, A., Prasad, C., Krishnan, K. & Chandrasharma, B. Age and anthropometric traits predict handgrip strength in healthy normals. *J Hand Microsurg* **2**, 58-61 (2010).

9 Ploegmakers, J. J., Hepping, A. M., Geertzen, J. H., Bulstra, S. K. & Stevens, M. Grip strength is strongly associated with height, weight and gender in childhood: a cross sectional study of 2241 children and adolescents providing reference values. *J Physiother* **59**, 255-261 (2013).

10 Wang, Y.-C., Bohannon, R. W., Li, X., Sindhu, B. & Kapellusch, J. Hand-Grip Strength: Normative Reference Values and Equations for Individuals 18 to 85 Years of Age Residing in the United States. *Journal of Orthopaedic & Sports Physical Therapy* **48**, 685-693 (2018).

11 Schaefer, L., Plotnikoff, R. C., Majumdar, S. R., Mollard, R., Woo, M., Sadman, R. *et al.* Outdoor time is associated with physical activity, sedentary time, and cardiorespiratory fitness in youth. *J Pediatr* **165**, 516-521 (2014).

12 Oliveria, S. A., Saraiya, M., Geller, A. C., Heneghan, M. K. & Jorgensen, C. Sun exposure and risk of melanoma. *Arch Dis Child* **91**, 131-138 (2006).

13 Suppa, M. & Gandini, S. Sunbeds and melanoma risk: time to close the debate. *Current Opinion in Oncology* **31** (2019).

14 German Guideline Program in Oncology (GGPO). Evidence-based Guideline on Prevention of Skin Cancer. Version 1.1 – April 2014. AWMF registration number: 032/052GGPO. Guideline (Long Version).

15 Gandini, S., Sera, F., Cattaruzza, M. S., Pasquini, P., Picconi, O., Boyle, P. *et al.* Meta-analysis of risk factors for cutaneous melanoma: II. Sun exposure. *European journal of cancer (Oxford, England : 1990)* **41**, 45-60 (2005).

16 Autier, P. Sunscreen abuse for intentional sun exposure. *British Journal of Dermatology* **161**, 40-45 (2009).

17 Holman, C. D., Armstrong, B. K. & Heenan, P. J. Relationship of cutaneous malignant melanoma to individual sunlight-exposure habits. *Journal of the National Cancer Institute* **76**, 403-414 (1986).

18 Lawler, S., Sugiyama, T. & Owen, N. Sun exposure concern, sun protection behaviors and physical activity among Australian adults. *Cancer Causes & Control* **18**, 1009-1014 (2007).

19 Rastrelli, M., Tropea, S., Rossi, C. R. & Alaibac, M. Melanoma: epidemiology, risk factors, pathogenesis, diagnosis and classification. *In Vivo* **28**, 1005-1011 (2014).
