# Supplementary material for: Association between physical activity, grip strength and sedentary behaviour with incidence of malignant melanoma: results from the UK Biobank
Source: Br J Cancer. 2021 May 31;125(4):593–600. doi: 10.1038/s41416-021-01443-5 (PMC8368160; doi:10.1038/s41416-021-01443-5)
Supplement: Supplementary file 2 — Completed STROBE Checklist [file 41416_2021_1443_MOESM2_ESM.pdf]

STROBE Statement—Checklist of items that should be included in reports of ***cohort studies***

	Item No	Recommendation
**Title and abstract**	1	(*a*) Indicate the study's design with a commonly used term in the title or the abstract<br>*The abstract describes the study design as "prospective cohort study" and names the study (UK Biobank) used for the research*
		(*b*) Provide in the abstract an informative and balanced summary of what was done and what was found<br>*The abstract describes methods and findings*
**Introduction**		
Background/rationale	2	Explain the scientific background and rationale for the investigation being reported<br>*The background and rationale are described in the "Introduction" section*
Objectives	3	State specific objectives, including any prespecified hypotheses<br>*The objective of the study is described in the "Introduction" section (last sentence)*
**Methods**		
Study design	4	Present key elements of study design early in the paper<br>*The study design is described in the section "Study population and data collection" (first subsection in the Methods section)*
Setting	5	Describe the setting, locations, and relevant dates, including periods of recruitment, exposure, follow-up, and data collection<br>*This information is described in the sections "Study population and data collection" and "Cohort follow-up and ascertainment of malignant melanoma cases"*
Participants	6	(*a*) Give the eligibility criteria, and the sources and methods of selection of participants. Describe methods of follow-up<br>*Eligibility criteria is described in the section "Study population and data collection" and Figure 1 shows a Flow chart. Follow-up is described in the section "Cohort follow-up and ascertainment of malignant melanoma cases".*
		(*b*) For matched studies, give matching criteria and number of exposed and unexposed<br>*Not applicable*
Variables	7	Clearly define all outcomes, exposures, predictors, potential confounders, and effect modifiers. Give diagnostic criteria, if applicable<br>*Exposures are described in the section "Assessment of physical activity, grip strength, and sedentary behaviour". Potential confounders are described in the section "Covariables" as well as in the Appendix S1. Outcomes are described in the section "Cohort follow-up and ascertainment of malignant melanoma cases"*
Data sources/ measurement	8*	For each variable of interest, give sources of data and details of methods of assessment (measurement). Describe comparability of assessment methods if there is more than one group<br>*Exposures are described in the section "Assessment of physical activity, grip strength, and sedentary behaviour". Outcomes are described in the section "Cohort follow-up and ascertainment of malignant melanoma cases"*
Bias	9	Describe any efforts to address potential sources of bias<br>*Adjustment methods and sensitivity analyses to address potential sources of bias are described in the section „Statistical Analysis"*
Study size	10	Explain how the study size was arrived at

		The study sample is described in the section "study population and data collection" and in the Flow chart (Figure 1)
Quantitative variables	11	Explain how quantitative variables were handled in the analyses. If applicable, describe which groupings were chosen and why<br>*Use of variables is described in the section "Statistical Analysis"*
Statistical methods	12	(*a*) Describe all statistical methods, including those used to control for confounding<br>*Statistical methods are discussed in the „Statistical Analysis" section.*
		(*b*) Describe any methods used to examine subgroups and interactions<br>Described in the section *„Statistical Analysis"*
		(*c*) Explain how missing data were addressed<br>*Described in the section "Study population and data collection" and „Statistical Analysis"*
		(*d*) If applicable, explain how loss to follow-up was addressed<br>*Not applicable*
		(*e*) Describe any sensitivity analyses<br>Described in the section *„Statistical Analysis"*
**Results**		
Participants	13*	(a) Report numbers of individuals at each stage of study—eg numbers potentially eligible, examined for eligibility, confirmed eligible, included in the study, completing follow-up, and analysed<br>*Described in the section "Study population and data collection" and shown in the Flow chart (Figure 1)*
		(b) Give reasons for non-participation at each stage<br>*See Flow chart (Figure 1)*
		(c) Consider use of a flow diagram<br>*See Flow chart (Figure 1)*
Descriptive data	14*	(a) Give characteristics of study participants (eg demographic, clinical, social) and information on exposures and potential confounders<br>*Age-standardised baseline characteristics are given in Tables 1, S5 and S6*
		(b) Indicate number of participants with missing data for each variable of interest<br>*Presented in Figure 1 (Flow chart) and Tables 1, S5 and S6*
		(c) Summarise follow-up time (eg, average and total amount)<br>*Presented in the first paragraph of the "Results" section*
Outcome data	15*	Report numbers of outcome events or summary measures over time<br>*Number of cases are reported in the first paragraph of the "Results" section and in Tables 2 and 3*
Main results	16	(*a*) Give unadjusted estimates and, if applicable, confounder-adjusted estimates and their precision (eg, 95% confidence interval). Make clear which confounders were adjusted for and why they were included<br>*Unadjusted and adjusted estimates are shown in Table 2 and are described in the "Results" section.*
		(*b*) Report category boundaries when continuous variables were categorized<br>*In the main analyses, exposures were not categorized.*
		(*c*) If relevant, consider translating estimates of relative risk into absolute risk for a meaningful time period<br>*Incidence rates per 10,000 person years are presented in the supplement (Table S4)*
Other analyses	17	Report other analyses done—eg analyses of subgroups and interactions, and sensitivity analyses

		Sensitivity analyses are described in the section "Statistical analyses" and their results are reported in the "Results" section
**Discussion**		
Key results	18	Summarise key results with reference to study objectives<br>*Results are summarised in the first paragraph of the "Discussion" section*
Limitations	19	Discuss limitations of the study, taking into account sources of potential bias or imprecision. Discuss both direction and magnitude of any potential bias<br>*Study limitations are discussed in the last paragraph of the "Discussion" section*
Interpretation	20	Give a cautious overall interpretation of results considering objectives, limitations, multiplicity of analyses, results from similar studies, and other relevant evidence<br>*Overall interpretation is given in the "Conclusion" section*
Generalisability	21	Discuss the generalisability (external validity) of the study results<br>*Generalisability is discussed in the "Discussion" section*
**Other information**		
Funding	22	Give the source of funding and the role of the funders for the present study and, if applicable, for the original study on which the present article is based<br>*Stated in the "Additional information" section*

*Give information separately for exposed and unexposed groups.

Note: An Explanation and Elaboration article discusses each checklist item and gives methodological background and published examples of transparent reporting. The STROBE checklist is best used in conjunction with this article (freely available on the Web sites of PLoS Medicine at http://www.plosmedicine.org/, Annals of Internal Medicine at http://www.annals.org/, and Epidemiology at http://www.epidem.com/). Information on the STROBE Initiative is available at http://www.strobe-statement.org.
